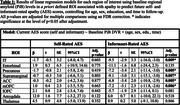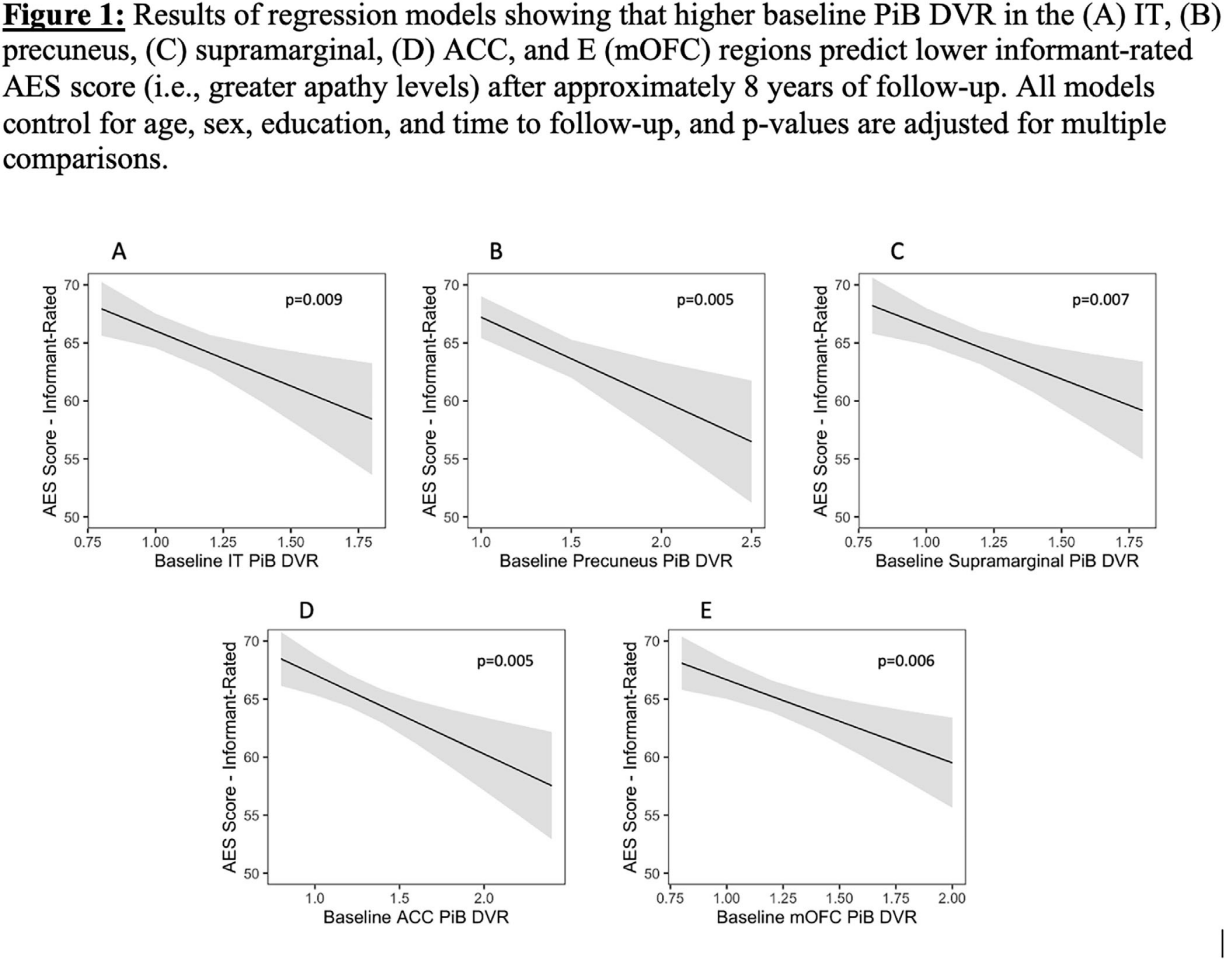# Baseline regional amyloid distribution in relation to subsequent self‐ and informant‐rated apathy scores in community‐dwelling older adults

**DOI:** 10.1002/alz.088000

**Published:** 2025-01-03

**Authors:** Catherine E Munro

**Affiliations:** ^1^ Harvard Medical School, Boston, MA, USA; Brigham and Women’s Hospital/Massachusetts General Hospital, Boston, MA USA

## Abstract

**Background:**

Apathy is a common neuropsychiatric symptom of Alzheimer’s disease (AD) and has been linked to greater levels of AD biomarkers (i.e., global cortical amyloid burden and regional tau levels) in preclinical individuals. However, it is unclear whether there are regional influences of amyloid on these relationships and if these relationships differ between self‐ and informant‐rated apathy. We sought to examine whether initial baseline levels of amyloid in brain regions linked to apathy in AD could predict self‐ and informant‐rated apathy at follow‐up.

**Method:**

202 participants who were cognitively unimpaired and nondepressed at baseline (mean age = 72.6) were included from the longitudinal Harvard Aging Brain Study cohort. MRI/PiB‐PET were performed at baseline and self‐ and informant‐rated apathy levels were obtained cross‐sectionally using Apathy Evaluation Scale (AES; lower scores indicating greater apathy) scores at their most recent visit (mean years follow‐up = 7.8). Regional PiB DVR values were calculated using FreeSurfer‐defined bilateral regions of interest (ROI) implicated in apathy: inferior temporal (IT), entorhinal, anterior cingulate (ACC), precuneus, supramarginal, and medial and lateral orbitofrontal (mOFC, lOFC) cortices, and the thalamus and amygdala. Separate linear regression models assessed whether regional baseline PiB levels predicted self‐ and informant‐rated apathy at follow‐up for each ROI, covarying for age, sex, education, and time‐to‐follow‐up, adjusted for multiple comparisons.

**Result:**

Higher baseline PiB levels in the IT, precuneus, supramarginal, ACC, and mOFC were associated with lower informant‐rated AES scores (indicating greater apathy) at follow‐up (Figure 1). Baseline PiB levels in any ROIs were not predictive of future self‐rated AES scores (Table 1). Results lost significance when examining only individuals who had a Clinical Dementia Rating score of 0 at follow‐up, indicating results were driven by those who progressed to cognitive impairment.

**Conclusion:**

In a cohort of older adults who were cognitively unimpaired and nondepressed at baseline, higher initial cortical amyloid levels in the IT, precuneus, supramarginal, ACC, and mOFC regions were associated with greater informant‐rated, but not self‐rated, apathy approximately 8 years later. These results shed light on the neurobiology of apathy in older individuals and preclinical AD and emphasize the importance of obtaining both self‐ and informant‐report when assessing new or worsening neuropsychiatric symptoms.